# Physical exercise is associated with a reduction in plasma levels of fractalkine, TGF-β1, eotaxin-1 and IL-6 in younger adults with mobility disability

**DOI:** 10.1371/journal.pone.0263173

**Published:** 2022-02-03

**Authors:** Parvin Kumar, Miranda Stiernborg, Anna Fogdell-Hahn, Kristoffer Månsson, Tomas Furmark, Daniel Berglind, Philippe A. Melas, Yvonne Forsell, Catharina Lavebratt

**Affiliations:** 1 Department of Molecular Medicine and Surgery, Karolinska Institutet, Stockholm, Sweden; 2 Center for Molecular Medicine, L8:00, Karolinska University Hospital, Stockholm, Sweden; 3 Department of Clinical Neuroscience, Karolinska Institutet, Stockholm, Sweden; 4 Centre for Psychiatry Research, Department of Clinical Neuroscience, Karolinska Institutet, Stockholm, Sweden; 5 Max Planck UCL Centre for Computational Psychiatry and Ageing Research, Berlin, Germany and London, United Kingdom; 6 Center for Lifespan Psychology, Max Planck Institute for Human Development, Berlin, Germany; 7 Department of Psychology, Uppsala University, Uppsala, Sweden; 8 Department of Public Health Sciences, Karolinska Institutet, Stockholm, Sweden; Holbaek Sygehus, DENMARK

## Abstract

Mobility disability (MD) refers to substantial limitations in life activities that arise because of movement impairments. Although MD is most prevalent in older individuals, it can also affect younger adults. Increasing evidence suggests that inflammation can drive the development of MD and may need to be targeted for MD prevention. Physical exercise has anti-inflammatory properties and has been associated with MD prevention. However, no studies to date have examined whether exercise interventions affect the peripheral inflammatory status in younger adults with MD. To this end, we used blood samples from young and middle-aged adults with MD (N = 38; median age = 34 years) who participated in a 12-week intervention that included aerobic and resistance exercise training. A pre-post assessment of inflammatory biomarkers was conducted in plasma from two timepoints, i.e., before the exercise trial and at follow-up (3–7 days after the last exercise session). We successfully measured 15 inflammatory biomarkers and found that exercise was associated with a significant reduction in levels of soluble fractalkine, transforming growth factor beta 1 (TGF-β1), eotaxin-1 and interleukin (IL) 6 (corrected α = 0.004). We also found significant male-specific effects of exercise on (i) increasing IL-16 and (ii) decreasing vascular endothelial growth factor-A (VEGF-A). In line with our results, previous studies have also found that exercise can reduce levels of TGF-β1, eotaxin-1 and IL-6. However, our finding that exercise reduces plasma levels of fractalkine in younger adults with MD, as well as the sex-dependent findings, have not been previously reported and warrant replication in larger cohorts. Given the suggested role of inflammation in promoting MD development, our study provides additional support for the use of physical exercise as a treatment modality for MD.

## Introduction

Mobility disability (MD) represents substantial limitations in performance of activities and participation in life situations, which arise because of movement impairments [1,2]. In the United States, about 10% of the population aged 18 years or older has been estimated to live with MD [3] and, in Sweden, the corresponding proportion has been estimated at 8% for those 16 to 84 years of age [4]. MD is associated with significant health care and social needs in affected individuals, making its prevention a public health concern [5]. Specifically, MD has been linked to a number of negative conditions, including living alone, being impoverished and poorly educated, having low health-related quality of life, and suffering from depression, anxiety, obesity, diabetes or cardiometabolic disorders [6,7]. Although MD is most prevalent in older individuals and those with chronic conditions such as musculoskeletal diseases and neurological disorders [8], MD can also affect younger adults [9]. Although the treatment options for MD are still limited, there is evidence supporting the use of physical activity interventions for preventing MD and for improving physical functioning in those already affected [10,11]. Nonetheless, more than half of the adults (18–64 years old) with MD in the United States have been reported to be physically inactive, and those who visited a health care professional often did not receive physical activity recommendations [6].

Currently, the presence of systemic inflammation has emerged as a possible biological mechanism that can drive the development of MD [12–17]; inflammation may, therefore, need to be targeted for MD prevention. Regular moderate-intensity aerobic exercise is known to improve cardiorespiratory fitness (CRF) and cardiometabolic biomarkers [18], and has well-documented anti-inflammatory properties [19,20]. Regular resistance training is also known to protect against cardiovascular diseases, although there is less consensus in the literature about its anti-inflammatory effects [21–23]. The anti-inflammatory properties of exercise are, at least partly, mediated by its effect on reducing stress hormones, myokines (from muscle cells), and inflammatory cytokines (from adipose tissue) [20]. However, to our knowledge, no studies have examined how exercise interventions, which couple aerobic with resistance exercise, affect inflammatory biomarkers in younger adults with MD. Moreover, sex-specific effects of physical exercise on immune activity markers are not well explored. To this end, we utilized data and samples from a published randomized control trial that studied the effects of two exercise-motivation intervention arms (i.e., smartphone-app versus supervised exercise) on levels of physical activity and CRF in younger adults (aged 18 to 45 years) with mild-to-moderate MD [24].

Specifically, trial participants in both arms were encouraged to engage in moderate-to-vigorous physical activity, which included both aerobic and resistance training, for a minimum of 30 minutes per day. The 12-week trial included a total of 110 participants at baseline and showed that both exercise interventions were equally effective in providing motivation for physical activity, enhancing CRF and improving body composition [24]. In the present study, we hypothesized that the exercise intervention would also have anti-inflammatory effects in this group of MD individuals. To examine this hypothesis, we collected venous blood samples from the first 38 participants of the trial and measured a panel of 24 immune activity plasma analytes (“inflammatory biomarkers”). Blood sampling was performed both prior to the first exercise session (i.e., baseline) and post-intervention (i.e., at 12 weeks follow-up). Post-intervention samplings were performed 3–7 days after the last exercise session to avoid measuring the acute effects of exercise.

Conclusively, since no studies to date have examined whether exercise interventions affect the peripheral inflammatory status in younger adults with MD, the objective of the present study was to conduct a pre-post exercise assessment of inflammatory biomarkers in plasma samples from young and middle-aged adults with MD. Here, we present the results from the successful pre-post measurements of 15 inflammatory biomarkers, some of which have not previously been well-studied in relation to physical exercise.

## Results

### Baseline characteristics of participants with mobility disability

The baseline characteristics of the study participants with MD (N = 38) are presented in Table 1. The participants reached moderate-to-vigorous physical activity levels corresponding to 43 (31–56), 48 (40–63) and 48 (35–61) min/day (median [25^th^-75^th^ percentiles]) as measured on the 1^st^, 6^th^ and 12^th^ week of the intervention, respectively. As shown in Table 2, there was no change in metabolic markers in the study participants following the exercise intervention.

**Table 1:** Baseline characteristics of the study participants with mobility disability (N = 38).

Baseline characteristics	
Age (years); median (IQR)	34 (28–39)
Male sex; n (%)	11 (29.0%)
Daily smoking; n (%)	2 (5.26%)
Alcohol use^a^; median (IQR)	3 (3–3)
BMI (kg/m^2^); median (IQR)	25.4 (22.3–30.9)
Fat mass (kg); median (IQR)	25.0 (17.1–34.5)
Fat-free mass (kg); median (IQR)	50.5 (45.3–59.5)
Ratio fat/fat-free mass; median (IQR)	0.472 (0.393–0.655)
VO_2_max categories^b^	
*Very low; n (%)*	9 (23.7%)
*Low; n (%)*	6 (15.8%)
*Somewhat low; n (%)*	6 (15.8%)
*Average; n (%)*	6 (15.8%)
*Somewhat high; n (%)*	5 (13.2%)
*High; n (%)*	4 (10.5%)
*Very high; n (%)*	2 (5.3%)
VO_2_max ([ml/min]/kg); median (IQR)	36.0 (28.0–40.0)

*Abbreviations*: BMI = Body Mass Index, VO_2_max = submaximal VO_2_max test, performed on a stationary bicycle, according to the Ekblom-Bak cycle ergometer test [25], and presented as ml/min per kg body weight.

^a^Alcohol use: 1 represents ≥ 4 times per week, 2 represents 2–3 times per week, 3 represents 2–4 times per month, 4 represents once per month, and 5 represents never.

^b^VO_2_max categories are based on reference values from ~25,000 Swedish males and females in working age (https://www.gih.se/ekblombaktest; see also Methods).

**Table 2:** Change (follow-up—Baseline, Δ) in metabolic markers during intervention.

Characteristics	Median (25^th^, 75^th^)
ΔVO_2_max ([ml/min]/kg)	2.0 (0.0, 4.0)
ΔVO_2_max categories	0.0 (0.0, 1.0)
ΔBMI (kg/m^2^)	-0.30 (-0.84, 0.18)
ΔFat mass (kg)	-0.89 (-2.4, 0.43)
ΔFat free mass (kg)	-0.65 (-1.7, 0.33)
ΔRatio fat/fat free mass	-0.015 (-0.040, 0.022)

*Abbreviations*: Δ = Follow-up—Baseline, BMI = Body Mass Index, VO_2_max = submaximal VO2max test, performed on a stationary bicycle, according to the Ekblom-Bak cycle, ergometer test [25], and presented as [ml/min]/kg.

### Bivariate correlations of inflammatory biomarkers and corrected threshold of significance

We successfully measured the following 15 inflammatory biomarkers in all study participants: (1) C-reactive protein (CRP), (2) eotaxin-1/CCL11, (3) soluble fractalkine/CX3CL1 (sFKN), (4) growth-regulated oncogene-alpha/CXCL1 (GRO-α), (5) soluble interleukin (IL)-2 receptor subunit alpha (sIL-2Rα), (6) IL-6, (7) IL-12/IL-23 subunit p40 (IL-12/IL-23p40), (8) IL-16, (9) IL-18, (10) serum amyloid A (SAA), (11) soluble intercellular adhesion molecule-1 (sICAM-1), (12) soluble vascular cell adhesion protein-1 (sVCAM-1), (13) transforming growth factor-beta 1 (TGF-β1), (14) tumor necrosis factor-related apoptosis-inducing ligand (TRAIL), and (15) vascular endothelial growth factor-A (VEGF-A). Bivariate correlation analyses of inflammatory analytes were conducted both at baseline and follow-up. At baseline, there were significant positive correlations (Spearman’s r ≥ 0.50) between levels of (a) GRO-α, TGF-β1 and VEGF-A, (b) SAA and CRP, (c) IL-18 and IL-12/IL-23p40, and (d) sICAM-1 and sVCAM-1 (S1A Fig). Three correlations with r ≥ 0.50 remained significant at follow-up: (a) SAA and CRP, (b) IL-18 and IL-12/IL-23p40, and (c) sICAM-1 and sVCAM-1 (S1B Fig). Thus, there were 12 independent analyte groups and the corrected significance was set at α = 0.004 (0.05/12).

### Exercise is associated with a significant reduction in sFKN, TGF-β1, eotaxin-1 and IL-6

**Fig 1:**
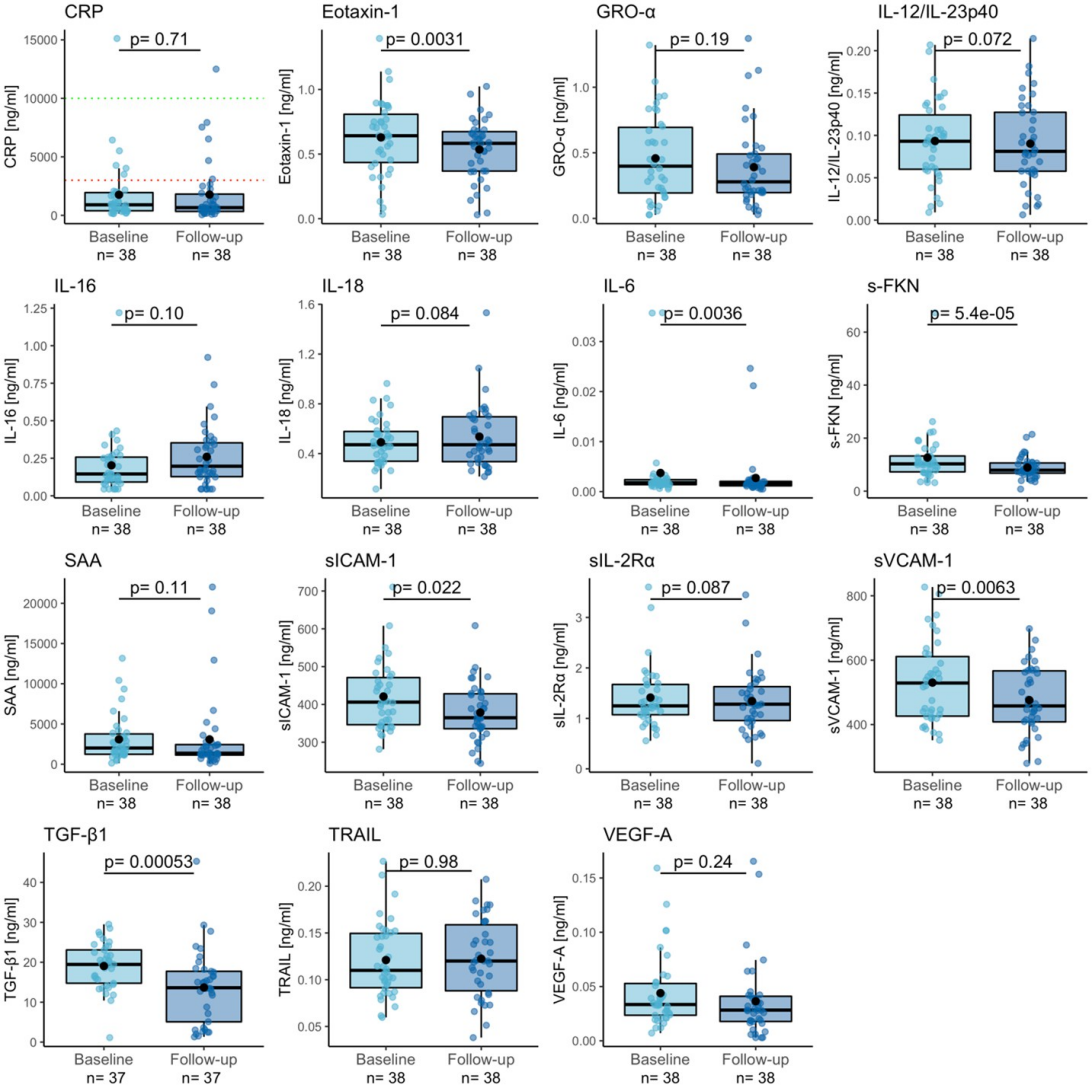
Exercise is associated with a reduction in sFKN, TGF-β1, eotaxin-1 and IL-6. The differences in plasma inflammatory biomarker levels between baseline and follow-up (i.e., before and after the 12-week physical exercise intervention program), were determined using Wilcoxon signed-rank tests. For CRP, the dotted red (3000 ng/ml) and the dotted green (10 000 ng/ml) lines represent the cut-offs for low-grade and mild-acute inflammation, respectively. Abbreviations: CRP = C-reactive protein, sFKN = soluble Fractalkine, GRO-α = Growth-regulated oncogene-alpha, IL-12/IL-23p40 = Interleukin (IL)-12/IL-23p40, IL-16 = Interleukin-16, IL-18 = Interleukin-18, sIL-2Rα = soluble Interleukin-2 receptor subunit alpha, IL-6 = Interleukin-6, SAA = serum amyloid A, sICAM-1 = soluble Intercellular adhesion molecule-1, sVCAM-1 = soluble Vascular cell adhesion molecule-1, TGF-β1 = Transforming growth factor beta 1, TRAIL = Tumor necrosis factor-related apoptosis-inducing ligand, VEGF-A = Vascular endothelial growth factor A.

Fig 1 shows the paired analyses of pre-post intervention changes in inflammatory biomarkers, which revealed significant exercise-associated reductions in levels of sFKN (p = 0.000054), TGF-β1 (p = 0.00053), eotaxin-1/CCL11 (p = 0.0031), and IL-6 (p = 0.0036). There was also a trend for a reduction in levels of sVCAM-1 (p = 0.0063). S1 Table shows the relationship between clinical baseline characteristics of the participants and (i) the baseline levels of these five inflammatory analytes, and (ii) the exercise-associated change (follow-up—baseline) in levels of these five inflammatory analytes. It should also be noted that although there were group-level exercise-associated reductions in levels of sFKN, TGF-β1, eotaxin-1, and IL-6, there was still variation in exercise response on the individual level (S2 Fig).

### Differences between males and females in inflammatory biomarker changes following exercise

**Fig 2:**
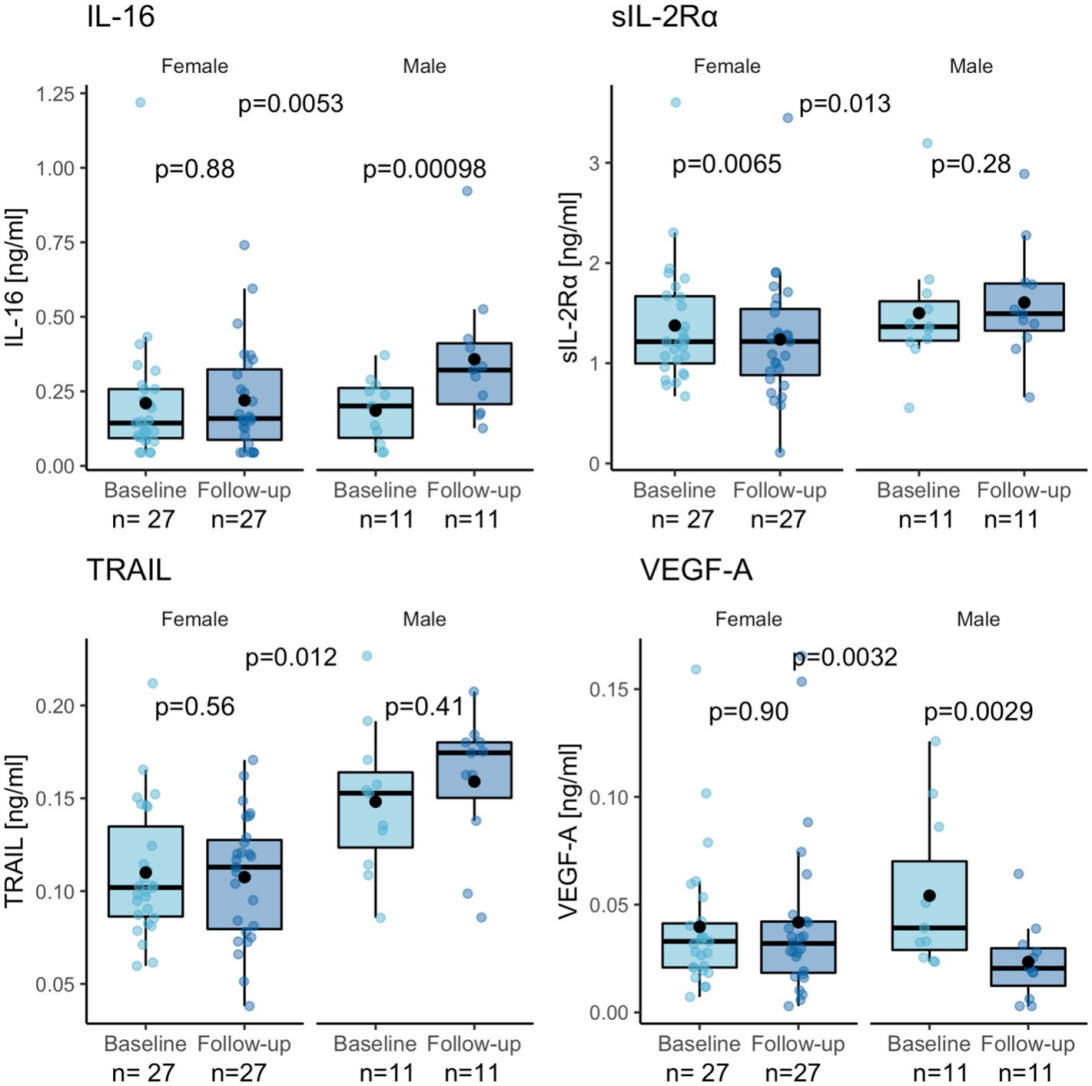
Sex-stratified analyses reveal differences in IL-16 and VEGF-A in males. Sex-stratified box plots of inflammatory biomarker levels before (baseline) and after (follow-up) the 12-week exercise intervention program. The differences in intervention response between sexes were evaluated with an ANCOVA model on analyte levels at follow-up adjusting for baseline analyte levels. The analytes with a difference in response between sexes at α = 0.05 are shown: IL-16 (p = 0.0053; partial eta^2^ = 0.202), sIL-2Rα (p = 0.013; partial eta^2^ = 0.164), TRAIL (p = 0.012; partial eta^2^ = 0.166), VEGF-A (p = 0.0032; partial eta^2^ = 0.223). The post-hoc pre-post differences in analyte levels for each sex were assessed using Wilcoxon tests (significance set at α = 0.004) and are presented under the corresponding sex. Abbreviations: IL-16 = Interleukin-16, sIL-2Rα = soluble Interleukin-2 receptor subunit alpha, TRAIL = Tumor necrosis factor-related apoptosis-inducing ligand, VEGF-A = Vascular endothelial growth factor A.

As shown in Fig 2, when we stratified for sex in our analyses, we found significant differences in pre-post change between sexes for IL-16 (p = 0.0053), sIL-2Rα (p = 0.013), TRAIL (p = 0.012), and VEGF-A (p = 0.0032). Post-hoc assessments showed that for males, but not for females, IL-16 levels increased (p = 0.00098) and VEGF-A levels decreased (p = 0.0029) following exercise. Conversely, females but not males, had a trend toward decreased levels of sIL-2Rα (p = 0.0065) following exercise.

## Discussion

The anti-inflammatory properties of exercise [19,20,26,27], which are observed “chronically”, i.e., following regular training [28], are of particular importance to MD because the presence of inflammation has emerged as a possible biological mechanism that can drive MD development [12–17]. In line with these “chronic” anti-inflammatory properties of regular exercise, our measurements (taken 3–7 days following the last exercise session of a 12-week intervention program) suggested that regular aerobic and resistance training led to an improvement in the peripheral inflammatory status of younger adults with mild-to-moderate MD. Specifically, following the exercise intervention, we found a significant reduction in plasma levels of four inflammatory biomarkers: (i) sFKN, (ii) TGF-β1, (iii) eotaxin-1 and (iv) IL-6. Moreover, (v) sVCAM-1 levels showed a trend for exercise-associated reduction, and sex-stratified analyses indicated different responses in males, compared to females, with post-exercise increases in IL-16 and decreases in VEGF-A levels in males only.

(i) Fractalkine (FKN) is a chemokine, primarily expressed by vascular endothelial cells, which exists in a cell-surface-bound form and a soluble form (sFKN). FKN is made soluble by metalloproteinase-dependent cleavage of its membrane-bound form [29]; a process that is induced by proinflammatory signals [30]. The soluble form, sFKN, shows strong chemoattractant activity towards T cells and monocytes bearing the fractalkine receptor (CX3CR1), and promotes leukocyte integrin activation. The membrane-bound FKN facilitates adhesion of leukocytes to the vascular endothelium and their migration into tissue [31]. Elevated plasma levels of sFKN have been associated with the development of metabolic syndrome [32], cardiovascular outcomes [33] and atherosclerosis (16). Thus, the reduction in plasma levels of sFKN in MD participants, which was observed in our study, may indicate an exercise-induced reduction in vascular inflammation. While aerobic exercise is known to reduce vascular inflammatory activity [23,34], only few studies have previously investigated the effect of physical exercise on levels of sFKN. These previous studies, however, found that 12 months of combined aerobic and resistance training, or 12 weeks of balance exercise programs, were associated with slightly elevated circulating levels of serum sFKN in either patients with type 2 diabetes and coronary artery disease, or in those without a diagnosis or with Parkinson’s disease [35,36]. Elevated levels of sFKN have also been found in serum of healthy individuals immediately following maximal exercise on a treadmill [37]. However, all of these previous studies measured sFKN levels in serum, which does not contain platelets, and FKN has a known role in mediating platelet adhesion [38]. Thus, since we measured sFKN levels using plasma, our findings of exercise-induced reductions in sFKN may indicate specificity to this liquid portion of blood, which warrants future investigation.
(ii) TGF-β1 is a multifunctional cytokine with an immune-modulatory role by inhibiting lymphocyte proliferation, stimulate IgA class switching of B cells and regulate tissue repair. Together with Il-6 it promotes the differentiation of pro-inflammatory CD4+ cells expressing IL-17. Higher plasma TGF-β1 levels have been associated with atherosclerosis, hypertension, hyperlipidemia and diabetes [39]. Moreover, there is evidence suggesting that increased TGF-β1 protein activity in skeletal muscle contributes to an impaired exercise response through inhibition of mitochondrial regulators and insulin signalling [40]. The expression of TGF-β1 is induced by mechanical loading in various tissues, including bone and cardiac tissue [41]. A previous study, using healthy individuals, found that TGF-β1 plasma levels increased transiently after two weeks of heavy resistance training, but that these chronic point-wise levels dropped below baseline following another two weeks of exercise [28]. These results are in agreement with our findings showing reduced TGF-β1 plasma levels after 12 weeks of regular exercise.
(iii) Eotaxin-1 is a chemokine expressed by the vascular smooth muscle cells located beneath the vascular endothelium, and also by, e.g., bronchial epithelial cells and dermal fibroblasts [42]. Eotaxin-1 expression is induced by proinflammatory cytokines (e.g., TNF-α) and it acts as a chemoattractant mainly for eosinophils, basophils, mast cells and Th2 lymphocytes. Higher plasma eotaxin-1 levels have been associated with asthma and allergy disorders but also neurodegeneration and accelerated aging [42]. In line with our findings of reduced eotaxin-1 levels following the 12-week exercise intervention, obese individuals who underwent eight-to-twelve week programs of aerobic and/or resistance exercise were also found to have reduced levels of eotaxin-1 [43–45].
(iv) IL-6 is a pleiotropic molecule with multiple immunologic and metabolic effects, which is both a pro-inflammatory cytokine and an anti-inflammatory myokine [46]. Higher levels of IL-6 have been found to predict, or to be associated with, physical impairment, disability and mortality in older persons [13,15,17,47,48]. Muscle-derived IL-6 is known to be released into the bloodstream during exercise, and is immediately followed by a transient increase in levels of anti-inflammatory IL-1 receptor antagonist (IL-1RA) and IL-10 [49–51]. Plasma levels of these three interleukins are back to pre-exercise levels in about 2 hours after a single exercise bout [52]. In line with our findings of reduced IL-6 levels following the 12-week exercise intervention, regular aerobic exercise has consistently been reported to decrease IL-6 levels in healthy adults [53,54]. The latter effect has also been proposed to be moderated by a genetic polymorphism in the *IL6* gene promoter, which regulates the beta-adrenergic activation of *IL6* transcription, in individuals with impaired glucose tolerance [55,56].
(v) One of the adhesion molecules measured, sVCAM-1, showed a trend for post-exercise reduction. sVCAM-1 levels correlate with levels of membrane-bound VCAM-1, which is expressed specifically by vascular endothelial and smooth muscle cells and is central to leukocyte–endothelial cell adhesion and leukocyte extravasation into the surrounding tissue [57,58]. Elevated levels of sVCAM-1 have been associated with vascular inflammation, endothelial dysfunction and atherosclerosis, and—similar to our findings—sVCAM-1 has been found to be reduced by low-to-moderate aerobic exercise in a variety of study populations, but without any effect of resistance exercise [34].

Although the mechanistic connections between fractalkine, TGF-β1, eotaxin-1 and IL-6 were not explored in our study, some assumptions can be made based on previous literature and our correlation analyses. For instance, TGF-β1 has previously been found to stimulate fractalkine expression in human cancerous cell lines and rat microglia [59,60]. This could indicate that the exercise-induced reductions in levels of fractalkine found in our study, are mediated by a primary effect of exercise on lowering TGF-β1 levels. However, we found no significant correlations between plasma fractalkine and TGF-β1 at either baseline or follow-up (S1 Fig), or between fractalkine level change (follow-up—baseline) and change in TGF-β1 (S3A Fig). Moreover, other studies found that TGF-β1 actually reduced fractalkine mRNA and protein expression in glioma cells [61], which supports a complex interplay between the levels of these two molecules that may be dependent on the tissue or disease of interest. Similarly, studies using smooth muscle cells or fibroblast cultures, found that TGF-β1 (together with IL-13) can stimulate the expression of eotaxin-1 [62,63]. Indeed, we detected a correlation between change in TGF-β1 and change in eotaxin-1 levels (r = 0.33, p = 0.046, S3B Fig). Along the same lines, cell culture experiments have demonstrated a crosstalk between TGF-β1 and IL-6, with TGF-β1 controlling aspects of early innate inflammation through promotion of IL-6 secretion and/or induction of IL-6 protein and mRNA levels [64–66], but without any significant correlations between these two molecules found in the plasma samples of our study. Interestingly, however, studies have found that plasma fractalkine levels correlate positively with plasma levels of IL-6 [67], which is in line with our correlation data for these two molecules that were significant at baseline (Spearman’s r = 0.42; S1 Fig), including the change in fractalkine that correlated with the change in IL-6 levels (r = 0.39, p = 0.015, S3C Fig). Indeed, previous studies using CD16+ monocytes suggested that membrane-bound fractalkine stimulates the production of IL-6 [68], providing a possible mechanistic link between the levels of these two molecules, which warrants additional investigation in physical exercise settings.

Besides our primary pre-post analyses, we also conducted sex-stratified analyses that revealed significant increases in IL-16, and significant reductions in VEGF-A, in male individuals only. IL-16 is known to stimulate the expression of pro-inflammatory cytokines [69] and has been associated with inflammatory diseases, such as rheumatoid arthritis [70]. By contrast, there are also reports suggesting that IL-16 may possess protective effects against other inflammatory disorders, such as atherosclerosis [71,72]. However, to our knowledge, no previous studies have reported sex-specific effects of physical exercise on IL-16 levels. VEGF-A, on the other hand, is produced by many different cell types, including vascular endothelial cells, macrophages and cardiomyocytes, and has a key role in mediating angiogenesis and vasodilation. Although VEGF-A is essential for the cardiovascular system, aberrantly elevated levels have been implicated in inflammatory processes of cardiovascular disease and pathology following atherosclerosis [73], as well as in inflammatory diseases, such as inflammatory bowel disease [74] and psoriasis-like disease [75]. Similar to our findings, plasma VEGF-A levels have previously been found to decrease for at least 2 hours, following a single bout of exercise, in healthy men [76]. By contrast, in overweight postmenopausal women, there was no effect of regular moderate-to-vigorous aerobic exercise on plasma levels of VEGF-A [77].

### Limitations

There are a number of limitations to this exploratory study that need to be acknowledged. (i) The low number of individuals included in our study (N = 38) render the significant findings preliminary and exploratory in nature, and all results need to be interpreted with caution prior to replication in additional MD cohorts. Moreover, for those analytes that we could not detect a significant pre-post exercise change, we cannot exclude the presence of true small effect sizes. Finally, the number of participants was even lower in the sex-stratified analyses. Hence, only relatively large effect sizes could have been detected. (ii) The intervention study, from which the current data and samples derived from, included an additional N = 50 individuals with MD who donated blood but were not included in our study. Comparisons of all known baseline characteristics between the two groups showed only slight sex differences, i.e., with some male overrepresentation in our group (p = 0.046, S2 Table). (iii) The study lacks long-term post-intervention data on MD status, repeated sampling without active intervention in MD, as well as a control group without MD. (iv) The blood samples were collected in a time window of 3–7 days after the last exercise session. The transient inflammatory-resembling responses often observed “acutely”, i.e., during or immediately after an exercise bout have been reported to last no longer than up to 24–36 h [37,52]. However, we cannot exclude the possibility of longer acute effects in some individuals. (v) As indicated by the levels of CRP in Fig 1, and in line with the hypothesis of inflammation in MD [12–17], a significant portion of the participants in our study had low-grade inflammation. However, CRP was not among the analytes that was significantly affected by exercise. Moreover, due to the lack of standardized reference levels for most of the remaining analytes, we cannot evaluate to which extent the participants’ levels deviated from normal ones. (vi) Although only few of the study’s MD participants reported a chronic disease, we cannot exclude the presence of undetected pathologies that could influence biomarker levels.

### Conclusion

Our findings support a beneficial role of regular moderate aerobic exercise, combined with resistance training, on both CRF [24] and peripheral inflammatory status in young and middle-aged adults with MD. Importantly, the post-exercise reduction in plasma sFKN levels has to our knowledge not previously been reported, but is in agreement with reduced plasma levels of vascular adhesion molecules reported by others [34], including the trend for reduced sVCAM-1 levels observed in our study. The post-exercise reductions that we found in plasma levels of TGF-β1, eotaxin-1 and IL-6, are in agreement with findings from other study groups [28,43–45,53,54], although there is to our knowledge no report on these markers in younger adults with MD. The male-specific exercise effects that we detected in levels of IL-16 and VEGF-A are also novel for MD subjects, although sex-specific effects on VEGF-A are supported by previous reports in non-MD groups [76,77]. However, given the aforementioned limitations of the present pilot study, future studies are warranted to confirm these findings and translate them into outcomes of clinical significance. Moreover, future studies need to take into consideration putative confounding parameters such as disease comorbidity and medication. Nonetheless, given the proposed role of inflammation in promoting MD development [12–17], our study provides support for the continued evaluation of physical exercise as a preventive and treatment modality for MD.

## Methods

### Participants

Study participants were the first 38 subjects (of the total N = 110) that participated in a 12-week parallel-group randomized controlled trial that took place between May 2018 –December 2018, and which was designed to compare the effects of an app-based program with a supervised exercise and health program on levels of physical activity and changes in CRF in individuals with mild-to-moderate MD [24]. As there was no difference in effects between the two motivation arms on amount of psychical activity or change in CRF or body composition, we did not account for intervention arm in this study. Participants were recruited from rehabilitation, primary care and occupational health care centres in Stockholm, Sweden. The eligibility criteria for study participation were as follows: male or female aged 18–45 years old with any mobility-related problems affecting everyday life, such as problems with transportation, work, dressing, and performing household or personal hygiene tasks, experienced during the three years prior to trial enrolment. Exclusion criteria included being bound to a wheelchair or having a medical condition preventing moderate-intensity walking (for a detailed description of the trial see also Berglind D *et al*. [78]). Only a few of the 38 participants in this study reported a chronic disease diagnosis. Of the 38, 19 were restricted in running, heavy lifts, and demanding sports, and 2 were restricted in household activities such as vacuum cleaning, walking in forest or gardening. Comparisons of the baseline characteristics of this study’s participants (N = 38) with the baseline characteristics of those individuals who contributed blood in the trial but were not included in the study (N = 50), are presented in S2 Table and show an overrepresentation of males in the present study (p = 0.046). All participants gave written informed consent before entering the trial, which was approved by the Ethical Review Board in Stockholm (Dnr: 2017/1206-31/1) and was registered in the ISRCTN registry (registration number: ISRCTN22387524). The present study was also approved by the same ethical committee. Moreover, all methods were carried out in accordance with relevant guidelines and regulations, and all experimental protocols were approved by the Ethical Review Board in Stockholm.

### Intervention, body composition and cardiorespiratory fitness (CRF)

The treatment arms were designed with an intrinsic motivation-to-change strategy in order to encourage participants to maintain sustained changes in moderate-to-vigorous physical activity and CRF [79]. In both treatment arms, participants were encouraged to engage in a minimum of 30 minutes of moderate-to-vigorous physical exercise every day, counting both aerobic and resistance exercise. The actual amount of physical activity was measured using Actigraph GT3X+ accelerometers, worn on the hip during all waking hours, for 7 consecutive days at each assessment. The assessments were at baseline, after 5 weeks and after 11 weeks of intervention. Management and analyses of the accelerometer data have been described elsewhere [24]. Briefly, valid measurements included ≥10 hours wear-time per day for ≥4 days. Wear-time and classification of bouts were computed using ActiLife v.6.13.3. Moderate-to-vigorous physical activity was defined as more than 3,208 counts per minute which in adults corresponds to a VO_2_/resting VO_2_ ≥ 3 or walking at ~4.5 km per hour [80,81]. The exercise programs were designed in collaboration with personal trainers and individualized based on baseline levels of CRF (VO_2_max), illness or physical symptoms. Body composition and CRF measurements in the study participants have been described in detail elsewhere [24]. In brief, fat mass (in kilograms; kg) and fat-free mass (kg) were measured through bioelectrical impedance [82] using an Omron body composition monitor (model HBF-511B-E/HBF-511T-E). Physical tests also included measurements such as height, weight and waist circumference. CRF measurements were performed using a submaximal VO_2_max test on a stationary bicycle according to the Ekblom-Bak cycle ergometer test [25] and are presented in relative numbers (ml/min per kg body weight). VO_2_max categories are based on age and sex-stratified reference values from ~25,000 Swedish males and females in working age (available at https://www.gih.se/ekblombaktest).

### Measurement of inflammatory biomarkers

Venous blood was collected from participants at baseline (prior to the first exercise session) and at the 12-week follow-up (3–7 days after the last exercise session) using lithium heparin tubes. Participants rested 40 minutes immediately prior to sampling. Refrigerated centrifugation was performed at 1,700 g for 20 minutes and plasma aliquots were stored at −80°C for 0.5–1.5 years until analysis. A total of 24 inflammatory/immune activity analytes were measured using the multiplex Meso Scale Discovery (MSD) Platform (Meso Scale Diagnostics; Maryland, USA) according to the manufacturer’s protocol at the MSD core facility, Center for Molecular Medicine, Stockholm. The analytes were the following: C-Reactive Protein (CRP), eotaxin-1/CCL11, soluble fractalkine/CX3CL1 (sFKN), growth-regulated oncogene-alpha (GRO-α), Interferon-γ (IFN-γ), interleukin (IL)-10, IL-12 subunit p40 (IL-12p40), IL-17A, IL-18, IL-1β, IL-2, IL-16, soluble interleukin-2 receptor subunit alpha (sIL2Rα), IL-6, monocyte chemoattractant protein 1 (MCP1), serum amyloid A (SAA), transforming growth factor beta 1, 2, and 3 (TGF-β1–3), tumor necrosis factor alpha (TNFα), TNF-related apoptosis-inducing ligand (TRAIL), soluble intercellular adhesion molecule 1 (sICAM-1), soluble vascular cell adhesion protein 1 (sVCAM-1), and vascular endothelial growth factor A (VEGF-A). The V-plex vascular injury panel 2 (MSD, Cat. no. K15198G) was used to measure CRP, SAA, ICAM-1 and VCAM-1, and the U-plex panels (MSD, Cat. nos. K15067L and K15241K) were used to measure the remaining analytes in multiplexes of 3, 7 and 10 analytes at experimental conditions designed by the manufacturer to give high sensitivity and avoid interference between analytes. Analytes with more than five samples under the lowest level of detection (LLOD) were excluded from further analyses, i.e., IL-17A, IL-1β, MCP1, TNFα, IFN-γ, IL-10, IL-2, TGF-β2, TGF-β3 (S3 Table). The LLOD for the remaining analytes were the following: On V-plex [ng/ml]; CRP = 3.34, SAA = 14.2, sICAM-1 = 1.86, sVCAM-1 = 9.27, and on U-plex [pg/ml]; eotaxin-1 = 9.12, sFKN = 103, GRO-α = 0.991, IL-6 = 0.502, IL-12/IL-23p40 = 1.37, IL-16 = 44.7, IL-18 = 0.438, sIL-2Rα = 81.2, TGF-β1 = 10.0, TRAIL = 2.84, VEGF-A = 2.91. All plasma samples were freeze-thawed twice. The manufacturer-delivered standard curve calibrators were measured in duplicates, and the patient samples were run in singlets based on empirically low coefficients of variation (CV) from MSD measurements in our lab. All samples were run on the same plate for each analyte and all standard curves had a robust correlation (r^2^ > 0.997). Within plate CVs were determined from the standard curve of each analyte and presented as median (IQR): CRP = 7.39% (6.10%–8.58%), SAA = 11.64% (10.56%–14.51%), sICAM-1 = 10.1% (7.31%–11.29%), sVCAM-1 = 9.49% (6.35%–10.94%), eotaxin-1 = 6.95% (4.18%–9.4%), sFKN = 2.46% (1.34%–3.63%), GRO-α = 5.31% (4.08%–5.84%), IL-6 = 6.3% (4.51%–8.28%), IL-12/IL-23p40 = 2.91% (1.6%–4.4%), IL-16 = 5.94% (4.52%–10.07%), IL-18 = 2.34% (1.39%–4.41%), sIL2Rα = 4.58% (3.97%–11.86%), TGF-β1 = 6.28% (3.47%–9.1%), TRAIL = 2.57% (1.49%–5.1%), VEGF-A = 2.74% (1.2%–4.75%). Data are presented as calculated values of undiluted plasma.

### Statistical analyses

To detect an effect of regular exercise on inflammatory biomarkers with the effect sizes previously reported [28,43,53,76,83], at an α = 0.05 and power = 0.80 for paired differences, a sample size of N = 10–35 individuals was estimated to be needed. Specifically, as several of the analytes measured by us were not previously included in studies of regular aerobic exercise, the sample size calculation was based only on eotaxin-1, IL-6, sICAM-1, sVCAM-1, TGF-β1 and VEGF-A. Group differences between those with and those without blood samples were tested using Mann-Whitney U tests (for continuous variables) and chi-square tests (for categorical variables). The bivariate correlations between clinical characteristics and the analyte levels were estimated with a non-parametric Spearman’s rank correlation test. Differences between baseline and follow-up were tested using non-parametric Wilcoxon tests and (as described in the main text) the corrected significance was set at α = 0.004 (0.05/12) since there were 12 independent analyte groups. Sex differences in pre-post analyte level response were tested using an ANCOVA model on the analyte levels at follow-up, adjusting for baseline levels. For analytes with a sex difference at α = 0.05, the post-hoc pre-post analyte level difference in each sex was assessed using Wilcoxon tests and with a significance set at α = 0.004. No possible outlier was excluded from the analyses. The detected pre-post differences were confirmed not to be biased by different time-of-the-day sampling at baseline compared to follow-up by removing from the analysis the nine samples with different pre-post time-of-the-day samplings (data not shown).

## Supporting information

S1 Fig Bivariate correlation analyses of inflammatory biomarkers. Correlation analyses between inflammatory biomarker levels at **(A)** baseline and **(B)** follow-up. Numbers represent Spearman’s rank correlation coefficients (r) and blue circles indicate a positive r different from zero at α = 0.01. Abbreviations: CRP = C-reactive protein, sFKN = soluble Fractalkine, GRO-α = Growth-regulated oncogene-alpha, IL-12/IL-23p40 = Interleukin (IL)-12/IL-23p40, IL-16 = Interleukin-16, IL-18 = Interleukin-18, sIL-2Rα = soluble Interleukin-2 receptor subunit alpha, IL-6 = Interleukin-6, SAA = serum amyloid A, sICAM-1 = soluble Intercellular adhesion molecule-1, sVCAM-1 = soluble Vascular cell adhesion molecule-1, TGF-β1 = Transforming growth factor beta 1, TRAIL = Tumor necrosis factor-related apoptosis-inducing ligand, VEGF-A = Vascular endothelial growth factor A.
(DOCX)

S2 Fig Individual presentation of differences in inflammatory biomarkers. The differences in inflammatory biomarker levels before (baseline) and after (follow-up) the 12-week exercise intervention program were determined using Wilcoxon signed-rank tests and using lines to indicate the intervention responses on the individual level. Abbreviations: CRP = C-reactive protein, sFKN = soluble Fractalkine, GRO-α = Growth-regulated oncogene-alpha, IL-12/IL-23p40 = Interleukin (IL)-12/IL-23p40, IL-16 = Interleukin-16, IL-18 = Interleukin-18, sIL-2Rα = soluble Interleukin-2 receptor subunit alpha, IL-6 = Interleukin-6, SAA = serum amyloid A, sICAM-1 = soluble Intercellular adhesion molecule-1, sVCAM-1 = soluble Vascular cell adhesion molecule-1, TGF-β1 = Transforming growth factor beta 1, TRAIL = Tumor necrosis factor-related apoptosis-inducing ligand, VEGF-A = Vascular endothelial growth factor A.
(DOCX)

S3 Fig Correlation between biomarkers, analysis on plasma level changes from baseline (bl) to follow up (fu). Spearman’s correlation analyses between **(A)** TGF-β1 and sFKN, **(B)** TGF-β1 and Eotaxin-1, and **(C)** sFKN and IL-6. Change is defined as level at follow up (fu) minus level at baseline (bl). Numbers represent Spearman’s rank correlation coefficients (r) and p-values. Abbreviations: sFKN = soluble Fractalkine, IL-6 = Interleukin-6, TGF-β1 = Transforming growth factor beta 1.
(DOCX)

S1 Table The relationship between levels of inflammatory biomarkers at baseline and clinical baseline characteristics, as well as the relationship between the change (follow-up—Baseline) in levels of inflammatory biomarkers and clinical baseline characteristics. (DOCX)

S2 Table Baseline characteristics of the study participants with mobility disability (N = 38) and comparisons with those MD individuals who contributed blood but were not included in this study (N = 50). (DOCX)

S3 Table Lower Level Of Detection (LLOD) values for all measured analytes. (DOCX)
