# Supplementary material for: Physical exercise is associated with a reduction in plasma levels of fractalkine, TGF-β1, eotaxin-1 and IL-6 in younger adults with mobility disability
Source: PLoS One. 2022 Feb 3;17(2):e0263173. doi: 10.1371/journal.pone.0263173 (PMC8812905; doi:10.1371/journal.pone.0263173)
Supplement: S1 Fig — Correlation analyses between inflammatory biomarker levels at (A) baseline and (B) follow-up. Numbers represent Spearman’s rank correlation coefficients (r) and blue circles indicate a positive r different from zero at α = 0.01. Abbreviations: CRP = C-reactive protein, sFKN = soluble Fractalkine, GRO-α = Growth-regulated oncogene-alpha, IL-12/IL-23p40 = Interleukin (IL)-12/IL-23p40, IL-16 = Interleukin-16, IL-18 = Interleukin-18, sIL-2Rα = soluble Interleukin-2 receptor subunit alpha, IL-6 = Interleukin-6, SAA = serum amyloid A, sICAM-1 = soluble Intercellular adhesion molecule-1, sVCAM-1 = soluble Vascular cell adhesion molecule-1, TGF-β1 = Transforming growth factor beta 1, TRAIL = Tumor necrosis factor-related apoptosis-inducing ligand, VEGF-A = Vascular endothelial growth factor A. (DOCX) [file pone.0263173.s001.docx]

**S1 Fig**


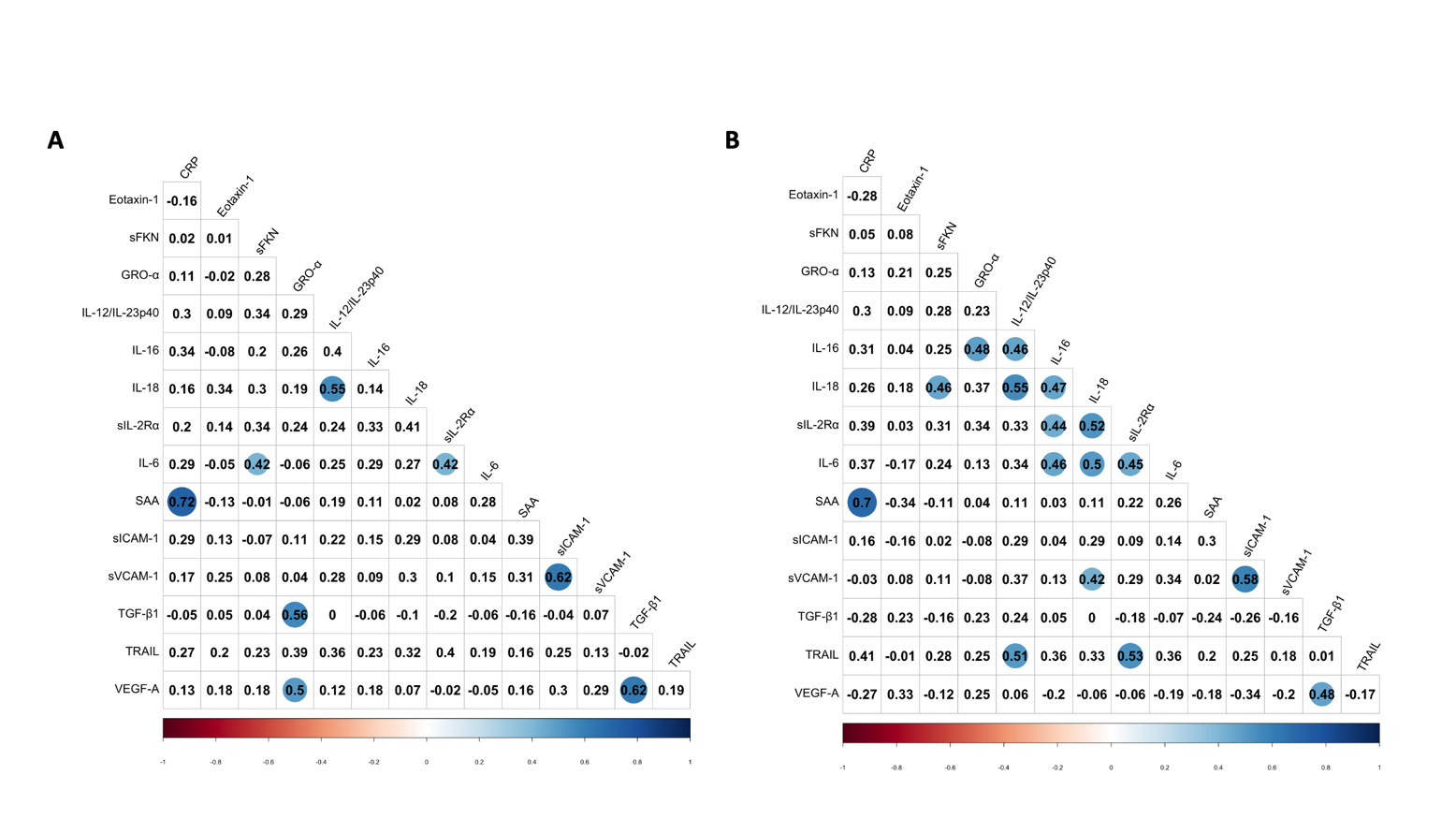


**Bivariate correlation analyses of inflammatory biomarkers.**

Correlation analyses between inflammatory biomarker levels at **(A)** baseline and **(B)** follow-up. Numbers represent Spearman’s rank correlation coefficients (r) and blue circles indicate a positive r different from zero at α = 0.01. Abbreviations: CRP = C-reactive protein, sFKN = soluble Fractalkine, GRO-α = Growth-regulated oncogene-alpha, IL-12/IL-23p40 = Interleukin (IL)-12/IL-23p40, IL-16 = Interleukin-16, IL-18 = Interleukin-18, sIL-2Rα = soluble Interleukin-2 receptor subunit alpha, IL-6 = Interleukin-6, SAA = serum amyloid A, sICAM-1 = soluble Intercellular adhesion molecule-1, sVCAM-1 = soluble Vascular cell adhesion molecule-1, TGF-β1 = Transforming growth factor beta 1, TRAIL = Tumor necrosis factor-related apoptosis-inducing ligand, VEGF-A = Vascular endothelial growth factor A.
