# Supplementary material for: Physical exercise is associated with a reduction in plasma levels of fractalkine, TGF-β1, eotaxin-1 and IL-6 in younger adults with mobility disability
Source: PLoS One. 2022 Feb 3;17(2):e0263173. doi: 10.1371/journal.pone.0263173 (PMC8812905; doi:10.1371/journal.pone.0263173)
Supplement: S2 Fig — The differences in inflammatory biomarker levels before (baseline) and after (follow-up) the 12-week exercise intervention program were determined using Wilcoxon signed-rank tests and using lines to indicate the intervention responses on the individual level. Abbreviations: CRP = C-reactive protein, sFKN = soluble Fractalkine, GRO-α = Growth-regulated oncogene-alpha, IL-12/IL-23p40 = Interleukin (IL)-12/IL-23p40, IL-16 = Interleukin-16, IL-18 = Interleukin-18, sIL-2Rα = soluble Interleukin-2 receptor subunit alpha, IL-6 = Interleukin-6, SAA = serum amyloid A, sICAM-1 = soluble Intercellular adhesion molecule-1, sVCAM-1 = soluble Vascular cell adhesion molecule-1, TGF-β1 = Transforming growth factor beta 1, TRAIL = Tumor necrosis factor-related apoptosis-inducing ligand, VEGF-A = Vascular endothelial growth factor A. (DOCX) [file pone.0263173.s002.docx]

**S2 Fig**


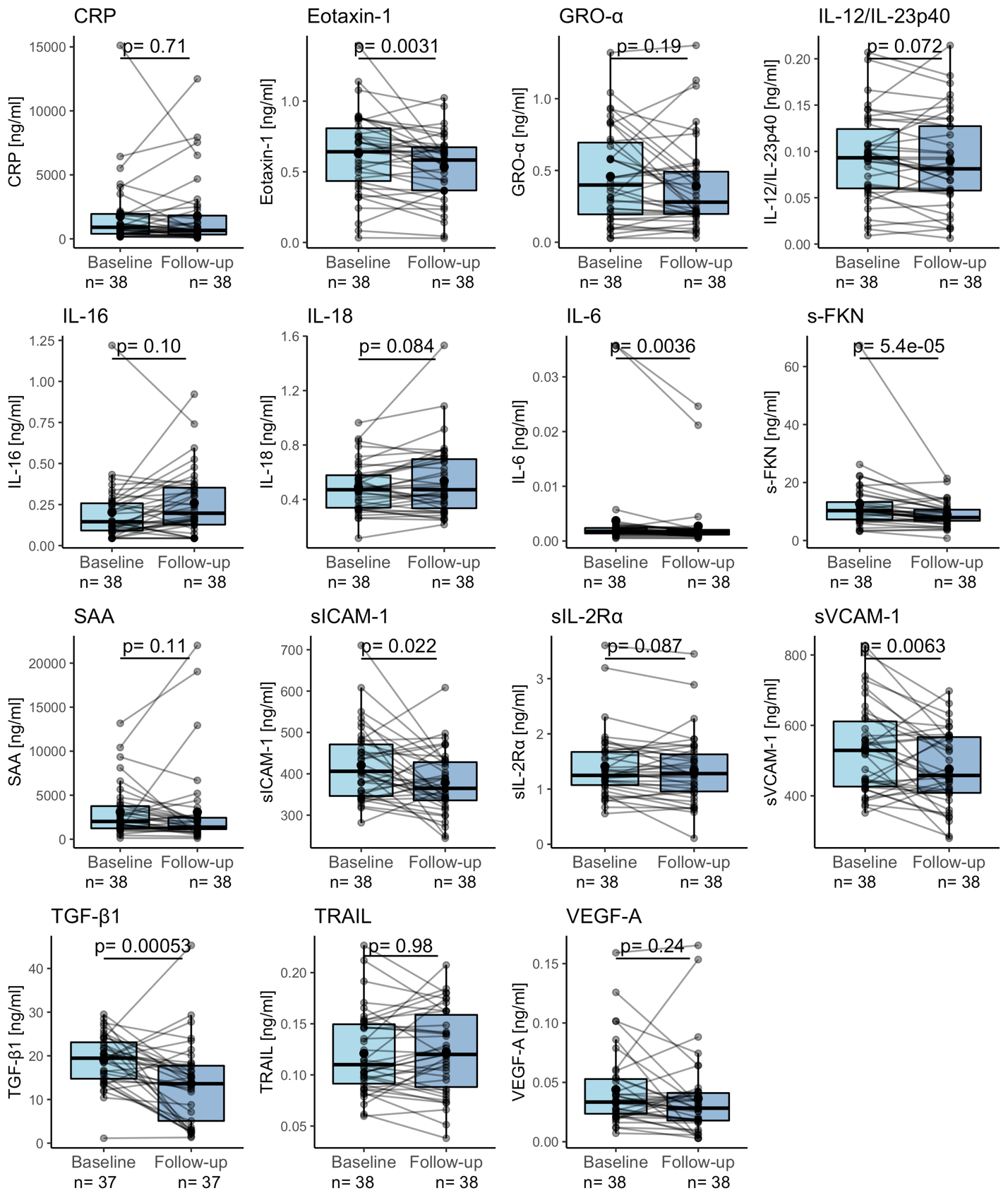


**Individual presentation of differences in inflammatory biomarkers.**

The differences in inflammatory biomarker levels before (baseline) and after (follow-up) the 12-week exercise intervention program were determined using Wilcoxon signed-rank tests and using lines to indicate the intervention responses on the individual level. Abbreviations: CRP = C-reactive protein, sFKN = soluble Fractalkine, GRO-α = Growth-regulated oncogene-alpha, IL-12/IL-23p40 = Interleukin (IL)-12/IL-23p40, IL-16 = Interleukin-16, IL-18 = Interleukin-18, sIL-2Rα = soluble Interleukin-2 receptor subunit alpha, IL-6 = Interleukin-6, SAA = serum amyloid A, sICAM-1 = soluble Intercellular adhesion molecule-1, sVCAM-1 = soluble Vascular cell adhesion molecule-1, TGF-β1 = Transforming growth factor beta 1, TRAIL = Tumor necrosis factor-related apoptosis-inducing ligand, VEGF-A = Vascular endothelial growth factor A.
