# Supplementary material for: Physical exercise is associated with a reduction in plasma levels of fractalkine, TGF-β1, eotaxin-1 and IL-6 in younger adults with mobility disability
Source: PLoS One. 2022 Feb 3;17(2):e0263173. doi: 10.1371/journal.pone.0263173 (PMC8812905; doi:10.1371/journal.pone.0263173)
Supplement: S3 Fig — Spearman’s correlation analyses between (A) TGF-β1 and sFKN, (B) TGF-β1 and Eotaxin-1, and (C) sFKN and IL-6. Change is defined as level at follow up (fu) minus level at baseline (bl). Numbers represent Spearman’s rank correlation coefficients (r) and p-values. Abbreviations: sFKN = soluble Fractalkine, IL-6 = Interleukin-6, TGF-β1 = Transforming growth factor beta 1. (DOCX) [file pone.0263173.s003.docx]

**S3 Fig**


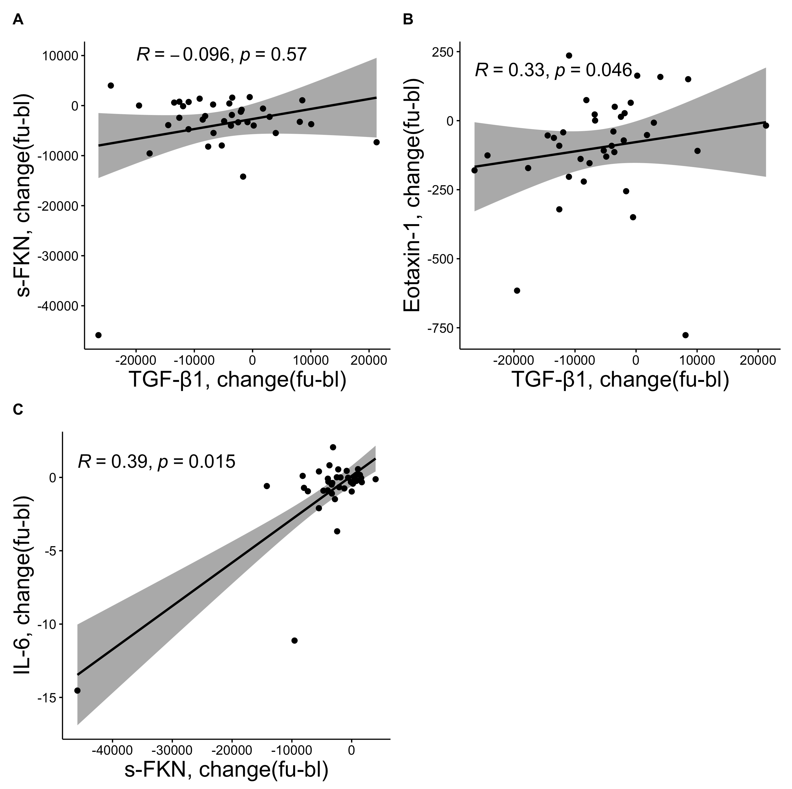


**Correlation between biomarkers, analysis on plasma level changes from baseline (bl) to follow up (fu).**

Spearman’s correlation analyses between **(A)** TGF-β1 and sFKN, **(B)** TGF-β1 and Eotaxin-1, and **(C)** sFKN and IL-6. Change is defined as level at follow up (fu) minus level at baseline (bl). Numbers represent Spearman’s rank correlation coefficients (r) and p-values. Abbreviations: sFKN = soluble Fractalkine, IL-6 = Interleukin-6, TGF-β1 = Transforming growth factor beta 1.
