# Supplementary material for: Physical exercise is associated with a reduction in plasma levels of fractalkine, TGF-β1, eotaxin-1 and IL-6 in younger adults with mobility disability
Source: PLoS One. 2022 Feb 3;17(2):e0263173. doi: 10.1371/journal.pone.0263173 (PMC8812905; doi:10.1371/journal.pone.0263173)
Supplement: S1 Table — (DOCX) [file pone.0263173.s004.docx]

**S1 Table. The relationship between levels of inflammatory biomarkers at baseline and clinical baseline characteristics, as well as the relationship between the change (follow-up – baseline) in levels of inflammatory biomarkers and clinical baseline characteristics.**

| *Baseline characteristics* | *Spearman’s rank correlation coefficient* | | | | | | | | | | |
| --- | --- | --- | --- | --- | --- | --- | --- | --- | --- | --- | --- |
|  | *Baseline* | | | | |  | *Change (follow-up – baseline)* | | | | |
|  | Eotaxin-1 | sFKN | IL-6 | TGF-β1 | ssVCAM-1 |  | Eotaxin-1 | sFKN | IL-6 | TGF-β1 | sVCAM-1 |
| Sex (male = 1, female = 0) | 0.315 | 0.114 | 0.013 | 0.336 | 0.050 |  | -0.230 | 0.019 | -0.050 | -0.044 | -0.029 |
| Age (years) | 0.186 | 0.056 | -0.039 | 0.045 | -0.222 |  | -0.245 | 0.126 | -0.115 | -0.024 | -0.028 |
| Daily smoking (yes = 1, no = 2) | -0.193 | 0.258 | 0.247 | -0.215 | 0.032 |  | -0.065 | -0.097 | -0.236 | -0.112 | 0.108 |
| Alcohol use^a^ | -0.310 | 0.075 | 0.031 | -0.166 | -0.259 |  | 0.005 | -0.202 | -0.233 | 0.161 | 0.050 |
| BMI (kg/m^2^) | -0.283 | -0.067 | 0.220 | -0.123 | -0.141 |  | -0.028 | 0.104 | 0.096 | 0.074 | 0.081 |
| Fat mass (kg) | -0.409 | -0.094 | 0.234 | -0.256 | -0.031 |  | 0.031 | 0.133 | 0.072 | 0.085 | -0.012 |
| Fat-free mass (kg) | 0.078 | 0.046 | -0.008 | 0.140 | -0.107 |  | -0.004 | 0.083 | 0.121 | 0.105 | 0.061 |
| Ratio fat mass/fat-free mass | -0.486** | -0.101 | 0.301 | -0.320 | 0.031 |  | 0.125 | 0.059 | -0.0071 | 0.084 | -0.026 |
| VO_2_max ([ml/min]/kg) | 0.573** | 0.130 | -0.201 | 0.072 | 0.211 |  | -0.022 | -0.107 | -0.012 | 0.034 | -0.149 |
| VO_2_max categorical^b^ | 0.469** | 0.093 | -0.189 | 0.053 | 0.234 |  | 0.034 | -0.072 | -0.041 | 0.086 | -0.220 |
| Eotaxin-1 (pg/ml) |  | 0.001 | -0.049 | 0.054 | 0.252 |  |  | 0.082 | 0.213 | 0.331 | -0.008 |
| sFKN (pg/ml) |  |  | 0.415* | 0.038 | 0.078 |  | -0.104 |  | 0.393 | -0.096 | 0.031 |
| IL-6 (pg/ml) |  |  |  | -0.061 | 0.154 |  | -0.218 | -0.383 |  | 0.272 | 0.061 |
| TGF-β1 (pg/ml) |  |  |  |  | 0.071 |  | -0.245 | 0.053 | -0.123 |  | -0.270 |
| sVCAM-1(pg/ml) |  |  |  |  |  |  | -0.010 | -0.074 | -0.029 | 0.135 |  |

^a^Alcohol use: 1 represents ≥ 4 times per week, 2 represents 2-3 times per week, 3 represents 2-4 times per month, 4 represents once per month, and 5 represents never.

^b^VO_2_max categories are based on reference values from ~25,000 Swedish males and females in working age.

*p ≤ 0.010; **p ≤ 0.004

*Abbreviations:*

sFKN= soluble Fractalkine

IL-6= Interleukin-6

TGF-β1= Transforming growth factor beta 1

sVCAM-1= soluble Vascular cell adhesion molecule-1

BMI= Body mass index
