# Supplementary material for: Physical exercise is associated with a reduction in plasma levels of fractalkine, TGF-β1, eotaxin-1 and IL-6 in younger adults with mobility disability
Source: PLoS One. 2022 Feb 3;17(2):e0263173. doi: 10.1371/journal.pone.0263173 (PMC8812905; doi:10.1371/journal.pone.0263173)
Supplement: S2 Table — (DOCX) [file pone.0263173.s005.docx]

**S2 Table. Baseline characteristics of the study participants with mobility disability (N = 38) and comparisons with those MD individuals who contributed blood but were not included in this study (N = 50).**

| Baseline characteristics | MD participants included in study | MD participants not included in study | Total MD participants^c^ | p-value |
| --- | --- | --- | --- | --- |
|  | n=38 | n=50 | n=88 |  |
| Age (years); median (IQR) | 34 (28-39) | 35 (31-40) | 35 (30-39) | 0.33 |
| Male sex; n (%) | 11 (29.0%) | 6 (12.0%) | 17 (19.3%) | 0.046 |
| Daily smoking; n (%) | 2 (5.26%) | 2 (4.08%) | 4 (4.55%) | 0.79 |
| Alcohol use^a^; median (IQR) | 3 (3-3) | 3 (3-4) | 3 (3-4) | 0.17 |
| BMI (kg/m^2^); median (IQR) | 25.4 (22.3-30.9) | 25.7 (23.4-27.9) | 25.6 (23.0-28.7) | 0.93 |
| Fat mass (kg); median (IQR) | 25.0 (17.1-34.5) | 26.4 (20.7-33.5) | 26.3 (18.3-34.2) | 0.61 |
| Fat-free mass (kg); median (IQR) | 50.5 (45.3-59.5) | 48.1 (45.7-51.0) | 49.0 (45.3-54.9) | 0.070 |
| Ratio fat/fat-free mass; median (IQR) | 0.472 (0.393-0.655) | 0.532 (0.405-0.725) | 0.492 (0.392-0.691) | 0.27 |
| VO_2_max categories^b^ |  |  |  | 0.60 |
| *Very low; n (%)* | 9 (23.7%) | 6 (12.5%) | 15 (17.4%) |  |
| *Low; n (%)* | 6 (15.8%) | 14 (29.2%) | 20 (23.3%) |  |
| *Somewhat low; n (%)* | 6 (15.8%) | 6 (12.5%) | 12 (14.0%) |  |
| *Average; n (%)* | 6 (15.8%) | 6 (12.5%) | 12 (14.0%) |  |
| *Somewhat high; n (%)* | 5 (13.2%) | 8 (16.7%) | 13 (15.1%) |  |
| *High; n (%)* | 4 (10.5%) | 7(14.6%) | 11 (12.8%) |  |
| *Very high; n (%)* | 2 (5.3%) | 1 (2.1%) | 3 (3.5%) |  |
| VO_2_max ([ml/min]/kg); median (IQR) | 36.0 (28.0-40.0) | 36.0 (30.0-41.0) | 36.0 (29.3-41.0) | 0.88 |

Group differences were tested using Mann-Whitney U tests (for continuous variables) and chi-square tests (for categorical variables).

*Abbreviations:*

BMI= Body Mass Index

MD = Mobility disability

VO_2_max= submaximal VO_2_max test, performed on a stationary bicycle, according to the Ekblom-Bak cycle ergometer test (see also Methods), and presented as ml/min per kg body weight.

^a^Alcohol use: 1 represents ≥ 4 times per week, 2 represents 2-3 times per week, 3 represents 2-4 times per month, 4 represents once per month, and 5 represents never.

^b^VO_2_max categories are based on reference values from ~25,000 Swedish males and females in working age (https://www.gih.se/ekblombaktest; see also Methods).

^c^In the original study by Berglind D. *et al.*, a total of N = 110 participants is described (see also Methods). However, N = 22 participants did not contribute blood samples at baseline and/or follow-up and are not included here.
