# Supplementary material for: Physical exercise is associated with a reduction in plasma levels of fractalkine, TGF-β1, eotaxin-1 and IL-6 in younger adults with mobility disability
Source: PLoS One. 2022 Feb 3;17(2):e0263173. doi: 10.1371/journal.pone.0263173 (PMC8812905; doi:10.1371/journal.pone.0263173)
Supplement: S3 Table — (DOCX) [file pone.0263173.s006.docx]

| Analysed^a^ | Analyte | Assay kit; Dilution | LLOD in study  (pg/ml) | LLOD according to manufacturer  (pg/ml) |
| --- | --- | --- | --- | --- |
| Yes | Eotaxin-1 | Flexible customized multiplex kit; 1:1 | 9.1 | 3.2 |
| Yes | sFKN | Flexible customized multiplex kit; 1:1 | 103 | 102 |
| Yes | GRO-α | Flexible customized multiplex kit; 1:1 | 0.99 | 0.25 |
| Yes | IL-6 | Flexible customized multiplex kit; 1:1 | 0.50 | 0.33 |
| Yes | IL-12/IL-23p40 | Flexible customized multiplex kit; 1:1 | 1.4 | 2.8 |
| Yes | IL-16 | Flexible customized multiplex kit; 1:1 | 44 | 6.6 |
| Yes | IL-18 | Flexible customized multiplex kit; 1:1 | 0.44 | 2.5 |
| Yes | sIL-2Rα | Flexible customized multiplex kit; 1:1 | 81 | 10 |
| Yes | TRAIL | Flexible customized multiplex kit; 1:1 | 2.8 | 0.66 |
| Yes | VEGF-A | Flexible customized multiplex kit; 1:1 | 2.9 | 2.0 |
| Yes | TGF-β1 | Flexible customized multiplex kit; 1:2 | 10 | 9.1 |
| Yes | CRP | Validated multiplex kit; 1:1000 | 3340^b^ | 1330^b^ |
| Yes | SAA | Validated multiplex kit; 1:1000 | 14200^b^ | 10900^b^ |
| Yes | sICAM-1 | Validated multiplex kit; 1:1000 | 1860^b^ | 1940^b^ |
| Yes | sVCAM-1 | Validated multiplex kit; 1:1000 | 9270^b^ | 6000^b^ |
| No | IFN-γ | Flexible customized multiplex kit; 1:1 | 2.8 | 1.7 |
| No | IL-1β | Flexible customized multiplex kit; 1:1 | 0.66 | 0.15 |
| No | IL-2 | Flexible customized multiplex kit; 1:1 | 2.5 | 0.70 |
| No | IL-10 | Flexible customized multiplex kit; 1:1 | 0.11 | 0.14 |
| No | IL-17A | Flexible customized multiplex kit; 1:1 | 1.1 | 2.1 |
| No | MCP-1 | Flexible customized multiplex kit; 1:1 | 2.2 | 0.74 |
| No | TNF-α | Flexible customized multiplex kit; 1:1 | 0.73 | 0.51 |
| No | TGF-β2 | Flexible customized multiplex kit; 1:2 | 2.7 | 2.5 |
| No | TGF-β3 | Flexible customized multiplex kit; 1:2 | 1.9 | 1.4 |

**S3 Table. Lower Level Of Detection (LLOD) values for all measured analytes.**

^a^: to be analyzed, an analyte had to have a maximum of 5 samples below LLOD

^b^: referring to undiluted plasma

Manufacturer: Meso Scale Discovery

CRP = C-reactive protein, sFKN = soluble Fractalkine, GRO-α = Growth-regulated oncogene-alpha, IFN-γ= Interferon gamma, IL-1β = Interleukin-1 beta, IL-2= Interleukin-2, IL-6 = Interleukin-6, IL-10= Interleukin-10, IL-12/IL-23p40 = Interleukin (IL)-12/IL-23p40, IL-16 = Interleukin-16, IL-17A= Interleukin-17A, IL-18 = Interleukin-18, sIL-2Rα = soluble Interleukin-2 receptor subunit alpha, MCP-1=Monocyte chemoattractant protein-1, SAA = serum amyloid A, sICAM-1 = soluble Intercellular adhesion molecule-1, sVCAM-1 = soluble Vascular cell adhesion molecule-1, TGF-β1 = Transforming growth factor beta 1, TGF-β2 = Transforming growth factor beta 2, TGF-β3 = Transforming growth factor beta 3, TNF-α = tumor necrosis factor α, TRAIL = Tumor necrosis factor-related apoptosis-inducing ligand, VEGF-A = Vascular endothelial growth factor A.
